# Assessing pragmatics in early childhood with the Language Use Inventory across seven languages

**DOI:** 10.3389/fpsyg.2023.1169775

**Published:** 2023-06-20

**Authors:** Diane Pesco, Daniela K. O’Neill

**Affiliations:** ^1^Department of Education, Concordia University, Montreal, QC, Canada; ^2^Department of Psychology, University of Waterloo, Waterloo, ON, Canada

**Keywords:** language development, pragmatics, social communication, parent report, Language Use Inventory, LUI, language assessment, cross-linguistic

## Abstract

The Language Use Inventory (LUI) is a parent-report measure of the pragmatic functions of young children’s language, standardized and norm-referenced in English (Canada) for children aged 18–47 months. The unique focus of the LUI, along with its appeal to parents, reliability and validity, and usefulness in both research and clinical contexts has prompted research teams globally to translate and adapt it to other languages. In this review, we describe the original LUI’s key features and report on processes used by seven different research teams to translate and adapt it to Arabic, French, Italian, Mandarin, Norwegian, Polish, and Portuguese. We also review data from the studies of the seven translated versions, which indicate that all the LUI versions were reliable and sensitive to developmental changes. The review demonstrates that the LUI, informed by a social-cognitive and functional approach to language development, captures growth in children’s language use across a range of linguistic and cultural contexts, and as such, can serve as a valuable tool for clinical and research purposes.

## Introduction

This mini-review considers seven studies aimed at translating and adapting the original English version of the Language Use Inventory (LUI; O’Neill, 2009) – a parent-report measure – to Arabic-Saudi Najdi dialect (AlKadhi, 2015), Canadian French (Pesco and O’Neill, 2016), Italian (Longobardi et al., 2017, 2021), Mandarin Chinese (Qian et al., 2022), Norwegian (Helland and Møllerhaug, 2020), Polish (Bialecka-Pikul et al., 2019), and European Portuguese (Guimarães et al., 2013; Guimarães and Cruz-Santos, 2020). The LUI is a standardized and norm-referenced parent questionnaire designed to specifically assess pragmatics for children aged 18–47 months old. The LUI asks parents about how their child is using language, including for what purposes, types of questions and comments, and how they adapt their communication to context (O’Neill, 2007, 2009). Its completion by parents is grounded on the premise that they are ideally suited to observe these early abilities in diverse contexts, and on evidence that parents are accurate reporters of children’s language production (Fenson et al., 2007) and that their reports are comparable in accuracy to screenings for language disorder carried out by trained examiners (So and To, 2022). Pragmatics is also referred to in the literature as social communication (e.g., Dillon et al., 2021) and implicated in the category of social (pragmatic) communication disorder (SPCD) introduced in the last edition of the Diagnostic and Statistical Manual of Mental Disorders (DSM-5; American Psychiatric Association [APA], 2013). As the use and differentiation of these terms is still evolving, we will continue to use the term pragmatics here.

We begin with key information about the original LUI (O’Neill, 2009) to set the stage for our review of seven studies of the LUI’s development in other languages, in terms of (a) the procedures used to translate and adapt the LUI to specific linguistic and cultural contexts, (b) the reliability of the translations, and (c) the translations’ developmental sensitivity, drawn from patterns of children’s LUI scores by age and gender across the translations.

## The Language Use Inventory

### Design of the LUI

The LUI is organized to three parts and 14 subscales, as shown in Table 1. Part 1, comprised of two subscales (A and B), focuses on children’s gestures. While Part 1 does not address language use *per se* and consequently does not figure into the LUI Total Score, it allows parents an opportunity to respond affirmatively even if a child produces only a few words. Parts 2 and 3, which comprise the LUI Total Score, ask parents to reflect on how their child uses language in daily life (e.g., “Your child’s requests for help”) and roughly follow a developmental sequence (i.e., Part 2 asks about pragmatic functions realized through words and early word combinations, while Part 3 focuses on longer sentences). Across Parts 2 and 3, 10 subscales focus on children’s language use (161 items comprising the LUI Total Score), while two unscored subscales (E and L) survey children’s interests in play and conversation via open-ended questions. Norms for the original LUI with English-speaking children are monthly and were derived from 3,563 children residing in Canada (O’Neill, 2009).

**TABLE 1:** Cronbach’s alpha coefficients by LUI part and subscale (studies ordered by sample size).

Study	English *N* = 3,563 (norming)	Portuguese *N* = 1,555^a^ (norming)	Polish *N* = 256	French *N* = 242	Italian *N* = 190^b^	Mandarin *N* = 177	Norwegian *N* = 139	Arabic *N* = 134
**Age or age range of children**	**18–47 months**	**18–47 months**	**32 months^c^**	**18–47 months**	**18–47 months**	**18–47 months**	**18–47 months**	**24–41 months**
LUI parts and subscales^d^								
Part 1 Gestures	0.88	0.88	0.83	0.86	0.87	0.90	0.86	–
A Asking for something	0.89	0.88	0.84	0.86	0.86	0.92	0.88	–
B Getting someone to notice something^e^	0.53	0.38	0.49	0.52	0.63	0.74	0.74	–
Part 2 Words	0.95	0.94	0.89	0.94	0.94	0.94	0.94	0.93
C Types of words	0.93	0.93	0.88	0.93	0.94	0.94	0.93	–
D Requests for help	0.88	0.80	0.64	0.76	0.74	0.71	0.77	–
Part 3 Longer Sentences	0.99	0.99	0.98	0.99	0.99	0.99	0.99	0.98
F Getting someone to notice something^e^	0.81	0.76	0.69	0.73	0.76	0.75	0.81	–
G Questions/comments – things	0.90	0.91	0.85	0.90	0.92	0.94	0.88	–
H Questions/comments – self/others	0.98	0.98	0.95	0.98	0.97	0.98	0.98	–
I Talk in activities with others	0.93	0.93	0.85	0.92	0.91	0.93	0.93	–
J Teasing/sense of humour^d^	0.79	0.75	0.59	0.78	0.73	0.83	0.78	–
K Interest in words and language	0.86	0.86	0.78	0.86	0.81	0.92	0.84	–
M Adapting conversation to others	0.93	0.93	0.85	0.93	0.89	0.95	0.91	–
N Building longer sentences and stories	0.98	0.97	0.96	0.98	0.96	0.98	0.97	–
LUI Total Score, Parts 2 and 3	0.99	–	0.85	–	–	0.99	0.99	–

En-dashes, not reported in publication.

^a^Guimarães and Cruz-Santos (2020).

^b^Longobardi et al. (2017).

^c^For the LUI-Polish longitudinal study, alpha coefficients were calculated at 20, 32, and 44 months (see p. 2325 of their article); we took the midpoint.

^d^For a description of parts and subscales, see section “Design of the LUI.” Note that E and L are unscored subscales.

^e^As noted in the text, subscale B has low variance and only 2 items, contributing to its lower alpha values; F and J also have relatively small numbers of items (6 and 5, respectively) compared to the other subscales.

For the 10 scored subscales, parents are asked about a particular use of language and, for most subscale items, are provided with examples of what a child might say. For example, one item asking whether a child expresses a desire to do something on their own is accompanied by the examples “I want to do it” and “Me do it.” The examples are intended to support parents’ understanding of the questions and also make clear to parents that variations in the form of children’s utterances are allowed. Parents typically find the LUI easy to complete, likely due to its intentionally simple format (mainly yes/no questions) and focus on parents’ recent observations – factors that enhance the reliability of parent reports (Fenson et al., 2007). The LUI also avoids probing language use influenced by social and cultural conventions of politeness and/or appropriateness but prone to more variation across cultures such as saying “please” or “bye-bye” (Pesco and O’Neill, 2012; O’Neill, 2014). Instead, it emphasizes language use driven by advances in children’s social cognition (O’Neill, 2007), such as children’s growing awareness of their own and others’ mental states, differences that may exist between them, and how children may need to adapt their communication as a result.

The LUI’s directions to parents also make clear that they can respond affirmatively to an item regardless of language (or other communicative means such as sign language) used by the child. Additionally, the LUI (in English) includes a question asking parents to estimate how much of the time their child is regularly exposed to a language other than English (range 0–100%). Only children whose parents reported they were exposed to English 80% or more of their waking hours were included in the norming sample (O’Neill, 2009). Thus, a parent’s estimate can be considered by clinicians in deciding whether to apply the LUI’s norms or report results only descriptively.

### Psychometric properties of the LUI and use in clinical practice and research

The LUI has strong discriminative, predictive, and concurrent validity. To elaborate, O’Neill (2007) showed that the LUI classified children into two groups – language delay or impairment based on clinical assessments by speech-language pathologists versus typically developing – with over 95% accuracy (i.e., sensitivity and specificity were each 95.9%). Pesco and O’Neill (2012) examined the LUI’s predictive validity with 348 children from the LUI norming sample. It was found that for children assessed with the LUI between the ages of 24 and 47 months, LUI scores predicted their language and communication skills at ages 5–6 years (*M* age 5;6) assessed via a protocol that included standardized language measures and clinical history. The values were 81% for sensitivity and 93% for specificity, despite a time interval of up to 3 years between the LUI and follow-up measures at ages 5–6, a factor known to lower these values (So and To, 2022). Additionally, children who scored below an empirically derived cut-off on the LUI at ages 24–47 months were 27 times more likely to display language difficulties at ages 5–6 than children scoring above the cut-off. Children’s LUI scores also concur with direct measures such as the Communication and Symbolic Behavior Scales (O’Neill, 2009), observations of language use in laboratory settings (Abbot-Smith et al., 2015), and an SLP- and parent-report measure, the Functional Communication Classification System (Caynes et al., 2021). The LUI also has high test-retest reliability (O’Neill, 2007, 2009).

The LUI’s unique focus on language use in daily life, design features, and psychometric properties have led to its wide use globally and to its recommendation as a benchmark measure of pragmatics (e.g., Tager-Flusberg et al., 2009). Researchers working with both the English and translated versions have also found the LUI to be a highly valuable tool to describe strengths and weaknesses in pragmatics among diverse groups of children, such as children with autism (Qian et al., 2022) and their siblings (Miller et al., 2015), children who have experienced neglect (Di Sante et al., 2019), and children with complex disabilities (Foster-Cohen and van Bysterveldt, 2016), amongst others. It has also been used in intervention to set goals for children and monitor their progress (Foster-Cohen and van Bysterveldt, 2016).

While there is continued discussion of whether routine, universal screening of early language is advisable (see Sansavini et al., 2021; So and To, 2022), the discriminative and predictive validity studies of the LUI described above provide support for screening and monitoring children, particularly when a concern about pragmatic language use is present (see also Miller et al., 2015; Conti et al., 2020). Additionally, the LUI’s internal reliability and sensitivity to growth in children’s language use are each high; data relevant to these features are reported in later sections where they serve as a comparison point for findings from studies of the seven LUI translations.

## Research on translations of the LUI into other languages

The LUI’s assets have prompted researchers globally to translate it into other languages. There are, at the time of writing, 16 translations completed or in-progress according to the publisher’s website.^1^ Our review focuses on the translations of the LUI to Arabic-Saudi Najdi dialect (AlKadhi, 2015), Canadian French (Pesco and O’Neill, 2016), Italian (Longobardi et al., 2017), Mandarin Chinese (Qian et al., 2022), Norwegian (Helland and Møllerhaug, 2020), Polish (Bialecka-Pikul et al., 2019), and European Portuguese (Guimarães et al., 2013; Guimarães and Cruz-Santos, 2020), seven languages for which authors have disseminated their research findings. We report on these studies next, addressing in turn the procedures for translating and adapting the LUI; the reliability of the translated/adapted versions; and the patterns observed across language in the children’s LUI scores overall and for boys and girls separately, given sex differences noted in the original English LUI norming study that led to separate norms.

### Translation and adaptation processes

A number of procedures were used across the studies to ensure the translated LUI was consistent with the original measure yet adapted as needed to be appropriate to the linguistic and cultural context. First, all seven studies involved translations of the instructions to parents and all items from English to the target language (i.e., *forward translation*). The examples of what a child might say to realize a particular pragmatic function were also translated to reflect children’s utterances in the target language. The forward translations for all the LUI translations were carried out by native speakers of the target language with expertise in child language (either the principal researchers or research assistants), and were then reviewed by expert panels. Among the members of these expert panels were translators, other research team members, consulting researchers from relevant fields (e.g., linguists), and speech-language pathologists. For the LUI-Arabic (AlKadhi, 2015), LUI-Portuguese (Guimarães et al., 2013; Guimarães and Cruz-Santos, 2020), LUI-Italian (Longobardi et al., 2017), and LUI-Norwegian (Helland and Møllerhaug, 2020), *back translation* (i.e., translating material back to the original language to check for equivalence) followed. The remaining teams used only forward translation to avoid the risk of overly literal back translations and confounding true differences in meaning between the translation and original with differential quality of the forward and back translations (see Qian et al., 2022 for sources recommending this approach).

A third procedure reported in the studies relates to the instructions to parents for completing the LUI. In the seven studies we reviewed, these remained very close to the original. Occasionally, a team deemed it necessary to adapt the instructions. Bialecka-Pikul et al. (2019) explained that it is common for children acquiring Polish – a morphologically rich language – to truncate multimorphemic or multisyllabic words. The research team thus added instructions to the LUI-Polish to encourage parents to consider truncated forms as words (p. 2322). For the LUI-Arabic, the instructions to parents were intentionally written in Modern Standard Arabic, while the examples of what children might say were provided in the Saudi Najdi dialect. Other teams reported no or only minor adjustments to the instructions or the items. The (free) license from the publisher to translate the LUI does ask researchers to report all changes, however minor, and the reason for these, to allow readers and users to understand just what was changed from the original English LUI, and why.

Parents were also invited by some research teams to review the LUI translations for any final revisions needed. Guimarães et al. (2013) engaged ten parents in a think-aloud session to obtain their feedback on the wording of items and examples of children’s language use for the LUI-Portuguese items and Pesco and O’Neill (2016) invited three Canadian (Quebec) mothers with varying educational levels to complete the LUI-French and comment on whether it was clear, thorough (i.e., covered their child’s language uses adequately) and easy to complete. AlKadhi (2015), Longobardi et al. (2021), and Qian et al. (2022) also engaged Saudi, Italian, and Chinese mothers (respectively) in a similar process.

### Reliability

For all versions, data were obtained by having parents of children of different ages complete the LUI in the target language. The ages of the children varied, with some of the seven teams including parents of children from 18 to 47 months of age as for the norming study of the original LUI, and other teams sampling children only at selected ages within this period (see Table 1 and section “Developmental sensitivity”). The decision to sample only at selected ages was mainly due to limitations in resources available to the research teams for these initial studies and/or consideration of the ages of child health checks or immunization schedules in the country of interest. As Table 1 shows, the size of the samples was also dramatically different, with as many as 1,555 participants in a norming study of the LUI-Portuguese and as few as 134 participants in the pilot study of the LUI-Arabic.

To test for internal reliability, the seven research teams uniformly calculated Cronbach’s alpha. The values are reported in Table 1 by study, for the LUI’s three parts and subscales. The coefficients for Part 1 (the gesture subscales) range from 0.83 to 0.90 but were lower for subscale B, likely as it is comprised of just two items related to pointing and showing gestures whose use remains high at all ages (i.e., have low variance), influencing the alpha value. For Parts 2 and 3 (comprising the 10 expressive subscales used to calculate a child’s LUI Total Score), the coefficients range from 0.93 to 0.99, with one exception (LUI-Polish Part 2 = 0.89). Thus, the parts of the LUI demonstrated very good to excellent internal reliability. The reliability analyses also showed that there was little need to change items, resulting in maximum LUI Total Scores that are equivalent to the original (161) or differ at most by 2. The need for so few changes suggests that the uses of language addressed on the LUI are present in early childhood across the diverse linguistic and cultural contexts studied. This unanticipated finding could be partly attributed to the LUI’s focus on uses of language that reflect children’s developing social cognition rather than social and cultural conventions, as noted in the section “Design of the LUI.”

### Developmental sensitivity

**FIGURE 1:**
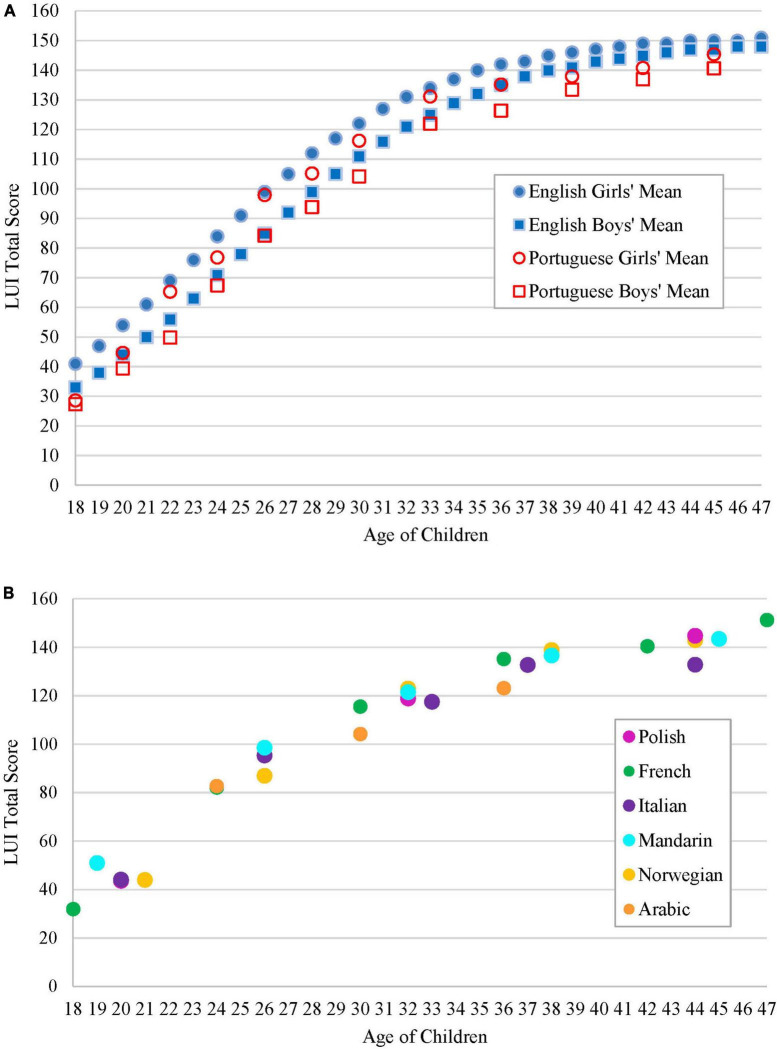
**(A)** Mean LUI Total Score by age and sex for the LUI (*N* = 3,563) and the LUI-Portuguese (*N* = 1,555) norming studies. **(B)** Mean LUI Total Score by age for six additional languages. For the LUI-Polish and LUI-French, means are for exact ages; for the remainder, the mean of the relevant age band is plotted.

For the LUI-Portuguese, Guimarães and Cruz-Santos (2020) reported data for 1,555 children from 18 to 47 months old at 2-month intervals (18, 20 months, etc.). Their data thus most resemble the English LUI norming data (O’Neill, 2009) where data were collected at every month. Figure 1A shows how children’s LUI Total Score compared across the two languages. It reveals a clear rise in scores at the younger ages for the two languages and for both boys and girls, followed by a more gradual rise or leveling off of scores at the older ages. The girls’ and boys’ means across the two languages are strikingly similar. Furthermore, higher scores for girls at some ages were observed in both samples and led to separate norms for boys and girls.

Of the six remaining studies, two reported data at selected ages, rather than at every month: namely at 18, 24, 30, 36, 42, and 47 months for the LUI-French, and at ages 20, 32, and 44 months for the LUI-Polish. The four other studies combined data from children in 6-month age bands, namely 18–23, 24–29, 30–35, 36–41, and 42–47 months for the LUI-Italian, LUI-Mandarin, and LUI-Norwegian, and from only the three middle age bands (i.e., 24–29, 30–35, 36–41 months) for the LUI-Arabic. Figure 1B shows the LUI Total Scores by age for these six translations. The data show that in the six studies, as in the two norming studies presented in Figure 1A, the LUI effectively captured developmental change in the 18–47-month period. First, growth in the LUI Total Score was observed, as evidenced by significant main effects for age in all the studies. Second, the reported *post hoc* comparisons of LUI Total Scores and/or of the LUI subscales showed that older children, on the whole, had significantly higher scores than younger children. These findings, drawn from the six studies with cross-sectional designs but also the one longitudinal study of the LUI-Polish, suggest that the LUI is sensitive to development. Furthermore, although the scores of older children at “near” ages (e.g., 36 vs. 42 months) were not always significantly different, they were in the expected direction (i.e., higher amongst the older children). It is also important to keep in mind that for children with difficulties in language or pragmatics, the onset of skills and subsequent growth in skills is likely to appear later than for typically developing children, thus resulting in significant differences even at the later ages.

Sex differences in the LUI Total Score observed in the norming studies for the original LUI and LUI-Portuguese were also found for the French, Italian, and Mandarin versions of the LUI: girls scored higher than boys, particularly at younger ages, while in the Polish longitudinal study, girls scored higher at both 30 and 44 months. Boys scored significantly higher than girls on only the LUI-Italian at older ages (i.e., in the 36–41 and 42–47 month groups). On the LUI-Norwegian and the LUI-Arabic, there were no significant differences between boys and girls on the LUI Total Score at any age, possibly due to the relatively smaller sample sizes per age group compared to the other studies.

## Discussion

This mini-review presents a first look at research conducted over the last decade on the development of translations of the LUI, a parent report measure of pragmatics, into seven different languages. By using forward translation and gathering feedback from multiple parties on the translation quality and the instructions for parents, the researchers developed versions that retain the original’s appeal to parents, reflect linguistic and cultural differences, and show comparable and high internal reliability across the studies. The results attest to the value of parent report and of the translations of the LUI in investigating early pragmatic abilities across many different languages.

The studies revealed an intriguing similarity across the different cultural and linguistic contexts in terms of growth in scores with age. Part of the reason for the similarity may be that the LUI, by design, avoids conventions that are known to be culturally specific (e.g., politeness markers) and judgments of “appropriateness,” and thus may reveal pragmatic functions that develop in early childhood across a wide range of contexts and regardless of the language a child is acquiring. The developmental sensitivity of each translation does not mean, however, that the pragmatic functions assessed by the LUI will emerge at precisely the same age across contexts (as one can see in Figure 1A, for example, the LUI-Portuguese scores appear slightly lower at most ages than the scores of English-speaking children in the LUI norming sample). Due to differences in the nature and size of the samples, and possibly environmental influences, one would expect some differences that research teams conducting translations could explore in more detail and/or larger studies.

### Limitations and future directions

A limitation of this mini-review is that we address only some qualities of the LUI in line with our goals, and available in existing published data. However, in the future, these seven research teams, and possibly others as published data becomes available, could be brought together to explore children’s scores on the LUI in more detail (e.g., subscale performance by sex and SES) as a means of further broadening our knowledge of early pragmatics and its development cross-linguistically. Clearly seven translations are only a fraction of possible translations and thus more extensive or different adaptations might be required by other languages. Additionally, the LUI translations have, so far, excluded children whose parents report over 20% exposure to another language. Given the prevalence of bi/multilingualism, it will be important in future work to assess how scores might differ at greater levels of bi/multilingualism, and in such cases whether the methodology of a single-percent parent estimate might need to be adjusted in a way that remains clinically practical.

Further study of performance on the subscales and/or items of the LUI and its translations of children in clinical groups (i.e., developmental language disorder; SPCD, autism spectrum disorder, deaf and hard-of-hearing) could also reveal distinct profiles of strengths and weaknesses in pragmatics amongst these groups or conversely, commonalities, cross-linguistically. Moreover, greater study of pragmatics alongside other aspects of language development (e.g., structural aspects) may aid in differentiating the impairments in SPCD and language disorders (Norbury, 2014). It is also possible that performance on a certain subset of scales may be more sensitive to, or more suited to, investigating various outcomes of interest in a particular language (Rints et al., 2015; Dockrell et al., 2022). Finally, as the LUI is translated and used in more diverse contexts (e.g., low- and middle-income countries), it could be of interest to explore further methodological and/or technological adaptations that could benefit parents with low-literacy levels.

## Author contributions

All authors listed have made a substantial, direct, and intellectual contribution to the work, and approved it for publication.

## Footnotes

1
https://languageuseinventory.com/translations

## Editor’s note

Maria-José Ezeizabarrena edited the article in collaboration with Melita Kovacevic, University of Zagreb, Zagreb, Croatia.
