# Erratum: Assessing pragmatics in early childhood with the Language Use Inventory across seven languages

**DOI:** 10.3389/fpsyg.2023.1258730

**Published:** 2023-08-09

**Authors:** Frontiers Production Office

**Affiliations:** Frontiers Media SA, Lausanne, Switzerland

**Keywords:** language development, pragmatics, social communication, parent report, Language Use Inventory, LUI, language assessment, cross-linguistic

Due to a publishing error, co-Editor accreditation needs to be amended. An Editor's Note has been added to the original article, as shown below.

**Editor's note**

Maria-José Ezeizabarrena edited the article in collaboration with Melita Kovacevic, University of Zagreb, Zagreb, Croatia.

The publisher apologizes for this mistake. The original article has been updated.

